# Impaired Wnt/β-catenin pathway leads to dysfunction of intestinal regeneration during necrotizing enterocolitis

**DOI:** 10.1038/s41419-019-1987-1

**Published:** 2019-10-03

**Authors:** Bo Li, Carol Lee, Marissa Cadete, Haitao Zhu, Yuhki Koike, Alison Hock, Richard Y. Wu, Steven R. Botts, Adam Minich, Mashriq Alganabi, Lijun Chi, Elke Zani-Ruttenstock, Hiromu Miyake, Yong Chen, Annika Mutanen, Bo Ngan, Kathene C. Johnson-Henry, Paolo De Coppi, Simon Eaton, Pekka Määttänen, Paul Delgado-Olguin, Philip M. Sherman, Augusto Zani, Agostino Pierro

**Affiliations:** 1 Translational Medicine Program, The Hospital for Sick Children, Toronto, ON M5G 1X8 Canada; 2 Division of General and Thoracic Surgery, The Hospital for Sick Children, Toronto, ON M5G 1X8 Canada; 3 Department of Pediatric Surgery, Children’s Hospital of Fudan University, 201102 Shanghai, China; 4 Cell Biology Program, The Hospital for Sick Children, Toronto, ON M5G 1X8 Canada; 5 Division of Pathology, The Hospital for Sick Children, Toronto, ON M5G 1X8 Canada; 6 UCL Great Ormond Street Institute of Child Health, London, WC1N 1EH UK; 7 Biology Department, Burman University, Lacombe, AB T4L 2E5 Canada; 8 Department of Molecular Genetics, University of Toronto, Toronto, ON M5S 1A8 Canada; 9 Heart & Stroke Richard Lewar Centre of Excellence, Toronto, ON M5S 3H2 Canada; 10 Division of Gastroenterology, Hepatology and Nutrition, The Hospital for Sick Children, Toronto, ON M5G 1X8 Canada; 11 Faculty of Medicine, Department of Laboratory Medicine and Pathobiology, University of Toronto, Toronto, ON M5S 1A8 Canada; 12 Faculty of Dentistry, University of Toronto, Toronto, ON M5G 1G6 Canada; 13 Developmental and Stem Cell Biology Program, The Hospital for Sick Children, Toronto, ON M5G 1X8 Canada; 14 Department of Surgery, University of Toronto, Toronto, ON M5S 1A8 Canada

**Keywords:** Intestinal diseases, Preclinical research

## Abstract

Necrotizing enterocolitis (NEC) is a devastating neonatal disease characterized by acute intestinal injury. Intestinal stem cell (ISC) renewal is required for gut regeneration in response to acute injury. The Wnt/β-catenin pathway is essential for intestinal renewal and ISC maintenance. We found that ISC expression, Wnt activity and intestinal regeneration were all decreased in both mice with experimental NEC and in infants with acute active NEC. Moreover, intestinal organoids derived from NEC-injured intestine of both mice and humans failed to maintain proliferation and presented more differentiation. Administration of Wnt7b reversed these changes and promoted growth of intestinal organoids. Additionally, administration of exogenous Wnt7b rescued intestinal injury, restored ISC, and reestablished intestinal epithelial homeostasis in mice with NEC. Our findings demonstrate that during NEC, Wnt/β-catenin signaling is decreased, ISC activity is impaired, and intestinal regeneration is defective. Administration of Wnt resulted in the maintenance of intestinal epithelial homeostasis and avoidance of NEC intestinal injury.

## Introduction

Necrotizing enterocolitis (NEC) is the most common gastrointestinal emergency in neonates and a major cause of death in preterm infants^1,2^. Despite recent advances in neonatal care, mortality from NEC remains high at 15–30%, demonstrating the need for innovative treatment strategies^3^. Furthermore, NEC pathogenesis and treatment strategy remains controversial in spite of various experimental and clinical trials being performed^4,5^.

The intestinal epithelial layer represents the first line of defense against luminal contents whereby specialized secretory cell types, such as Paneth cells, secrete antimicrobial factors to prevent tissue damage^6^. To maintain the integrity and viability of the epithelial layer, intestinal epithelial cells are in constant turnover and are replenished by intestinal stem cells (ISC) that express the Leucine-rich repeat-containing G-protein coupled receptor 5 (Lgr5)^7^. These cells are localized within the intestinal crypts and are critical for damage-induced intestinal regeneration^8^. We previously demonstrated that intestinal injury induced by maternal separation leads to expansion of ISC and increases cell proliferation, thereby preventing further intestinal damage^9^. ISC depletion correlates with severe gut damage during NEC development^10^, and dietary agents that promote ISC expansion can ameliorate NEC severity^11^. These findings highlight a critical link between ISC function and intestinal repair processes.

Proliferation and maintenance of ISC are controlled by the Wnt/β-catenin signaling pathway^12^. Wnt ligands are released by Paneth cells^13^ and stromal cells located underneath the epithelial surface including macrophages^14^, endothelial cells, and neurons^15^. Upon release, Wnt ligands activate low-density lipoprotein receptor-related protein 5/6 (LRP5/6) and Frizzled co-receptors to facilitate translocation of β-catenin to the nucleus, where it interacts with transcription factor 4 (TCF4) to maintain proliferation and differentiation of both stem cells and epithelial cells^15^. Disruption of the Wnt/β-catenin pathway by TCF4 knockout compromises cell proliferation in the neonatal small intestine, while constitutively active β-catenin stimulates cell proliferation^16,17^. A decrease in Wnt-producing Paneth cells^18^ and stromal cells^19–21^ is associated with experimental NEC. However, ISC impairment in NEC may be caused by defective Wnt signaling. Whether defective Wnt signaling underlies ISC impairment in NEC remains to be investigated.

Intestinal epithelial organoids are cultured and formed from intestinal crypts^22^ and have been used to study human development and various disease pathogeneses^23,24^. They were also been used to study NEC progression and to search for novel therapeutic treatments. Intestinal organoids were derived from wildtype and mutant mice and exposed to intestinal injury stimulators to establish NEC-like injury^25,26^. Similarly, intestinal organoids derived from human intestinal tissue were utilized as an ex vivo NEC model^27^. Recently, we have described the protocol to derive neonatal intestinal organoids from both mice and humans and found that these organoids maintain viability and respond to intestinal injury stress factors^28^. Intestinal organoids are useful and powerful tools to investigate NEC pathogenesis^25–28^. Intestinal organoids derived from infants undergoing surgery for NEC were capable of growing and differentiating into all relevant intestinal cell-type lineages^29^. Organoids derived from NEC tissue have the potential to enhance our understanding of the intestinal pathophysiology of NEC and hold promise for the development of future therapeutic treatments.

In our study, we hypothesized that the intestinal epithelium failed to regenerate during NEC intestinal injury due to the deficiency of endogenous Wnt. We also speculated that exogenous Wnt administration would promote intestinal regeneration and attenuate intestinal injury ex vivo in intestinal organoids and in vivo in neonatal mice exposed to experimental NEC.

## Methods and materials

### Animals and NEC model

All animal experiments were approved by the Animal Care Committee at The Hospital for Sick Children (no. 32238), and all methods were performed according to its guidelines and regulations. NEC was induced in neonatal C57BL/6 mixed sex mice from postnatal day 5–9 by gavage feeding of hyperosmolar formula (15 g Similac dissolved in 75 mL Esbilac; osmolality 849 mOsm/kg^30^), exposure to hypoxia (5% O_2_ for 10 min, three times daily), and oral LPS (4 mg/kg/day on day 6 and 7 only) for 4 days. Pups were randomly assigned to each of the experimental groups to eliminate potential litter effects. On postnatal days 6 and 7, mice received an intraperitoneal injection of phosphate-buffered saline (PBS, *n* = 14) or human recombinant Wnt7b (1 µg/pup, *n* = 11, Abcam, Cambridge, MA). Breastfed mice (*n* = 10), and breastfed mice injected with Wnt7b served as controls (*n* = 10). At postnatal day 9, survival pups were sacrificed, and ileum was harvested and fixed in 4% paraformaldehyde (10 pups for each group). Mice with GFP-labelled Lgr5 + ISC (*Lgr5-EGFP-IRES-creERT2*) were obtained from Jackson Laboratory (Sacramento, CA) to study intestinal stem cells after inducing NEC and Wnt7b administration (six pups for each group).

NEC induction was performed by the same researcher who was fully trained in this NEC model and developed expertise in pup mice handling. All the animals who died were examined within a maximum of 6 h from death and in no instances mortality appeared to be related to technical issues such as gastric perforation or delivery of formula into the lungs.

### Human small intestine

Ethical approval for this study was obtained from the Research Ethics Board of the Hospital for Sick Children, Toronto, Canada (protocol #1000056881). All methods performed in the study were carried out in accordance with the approved guidelines and regulations. Tissue analysis was done with approval from the Hospital for Sick Children and in accordance with anatomical tissue procurement guidelines. Except for the study principal investigator (A.P.) and study coordinator (M.C.), no study personnel analyzing these samples had access to personal identification information.

Human samples were obtained from the ileum of infants with NEC that were stored by the Division of Pathology. The ileum was resected during emergency laparotomy for “acute active NEC” (*n* = 5). The samples analyzed from all patients in this study were from areas of ileum not involved in necrosis (postmenstrual age, median 28, range 24–33 weeks). Age-matched “non-NEC” control samples (*n* = 5) were obtained from normal portions of ileum of infants undergoing surgery for congenital intestinal obstruction (median 33, range 30-36 weeks). All infants were premature and were operated on during the first 7 weeks of life.

In addition, to derive human intestinal organoids from normal portions of ileum, fresh human samples of ileum were obtained from premature infants undergoing laparotomy for resection of post-NEC intestinal strictures when inflammation of the ileum was not present (*n* = 5). Informed consent was obtained from all parents/legal guardians of the patients.

### Intestinal morphology analysis

Ileal tissue was embedded in paraffin, sectioned (5 µm) and counterstained with hematoxylin. Stained sections were assessed by three blinded investigators using an established NEC histopathological scoring system^31,32^. Mice with grade ≥ 2 were considered NEC positive.

### Tissue and cell immunostaining

Sections of terminal ileum were immunostained with primary antibodies for Ki67 (1:500) (Abcam, Cambridge, MA), green fluorescent protein (GFP) and β-catenin (Cell Signaling Technology, Danvers, MA), followed by secondary antibodies and DAPI (1:1000) (Vector Laboratories, Burlington, ON). For subsequent reactions, a streptavidin-biotin complex peroxidase kit (LASB + Kit, Dako, Denmark) was used. Slides were analyzed using a Nikon TE-2000 digital microscope equipped with a Hamamatsu C4742-80-12AG camera. Quantification was performed by three blinded investigators.

### Gene expression

Gene expression was quantified using RT-qPCR as previously described^9^. Data were analyzed using CFX Manager 3.1 (Biorad, Hercules, CA). Results are from three independent experiments each performed in triplicate. Expression levels were calculated by the ∆∆Ct method and normalized to reference housekeeping genes *Glyceraldehyde 3-phosphate dehydrogenase (GAPDH)* and *gene ribosomal protein large, P0 (RPLP0)*.

### Immunoblotting analysis

Protein expression was quantified using immunoblotting analysis as previously described^9^. The membrane was probed with primary antibodies for Wnt3, Wnt7b, and H3 (1:500) (Cell Signaling Technology, Danvers, MA) overnight at 4 °C and secondary antibodies (1:1000) at room temperature. Immuno-positive bands were detected using an ECL Plus kit (Invitrogen, Carlsbad, CA). Band intensities were determined using an Odyssey scanner (LI-COR Biosciences, Lincoln, NE). Densitometry ratios were calculated relative to levels of H3.

### Intestinal organoids

Intestinal organoids were cultured according to the protocol from StemCell Technologies (Cambridge, MA). Small intestine was harvested from p9 pups mice and cut into small segments. Intestinal crypts were isolated by digestion with Gentle Cell Dissociation Reagent (StemCell Technologies, Cambridge, MA) for 15 min and pelleted by centrifugation. Crypts were then re-suspended in Matrigel (Corning, New York) and transferred into 24-well plates. After polymerization, mouse IntestiCult organoid growth medium (StemCell Technologies, Cambridge, MA) supplemented with penicillin-streptomycin (100U/ml) was overlaid on the gel in each well. Organoids were maintained in a 37 °C incubator with the culture medium replaced every 2 days. Organoids were exposed to PBS, or Wnt7b (200 ng/ml in the culture medium) for 7 days. Control organoids were also grown in medium with and without Wnt, according to a previously published organoids culture medium protocol^30^. Organoids were imaged daily, and their surface area was calculated using Image J software. RNA was extracted with Trizol after removing culture medium. For immunofluorescence, organoids were fixed with 4% PFA for 30 min, permeabilized, and blocked for non-specific binding with 3% BSA prior to incubation with primary antibodies.

Human intestinal organoids were cultured as described above, with human IntestiCult organoid growth medium (StemCell Technologies, Cambridge, MA) and 10 μM Y-27632 (StemCell Technologies, Cambridge, MA), a ROCK inhibitor was added to the medium for primary culture.

### Statistics

Results are presented as means ± SD for normally distributed data, or median and range for non-normally distributed data (Kolmogorov-Smirnov test). Groups were compared using one-way ANOVA with Bonferroni-corrected post-hoc tests as appropriate. Survival curves were compared using the log-rank test. *p* < 0.05 was considered statistically significant.

## Results

### Intestinal epithelial regeneration and intestinal stem cells are impaired in both mouse and human NEC

**Fig. 1:**
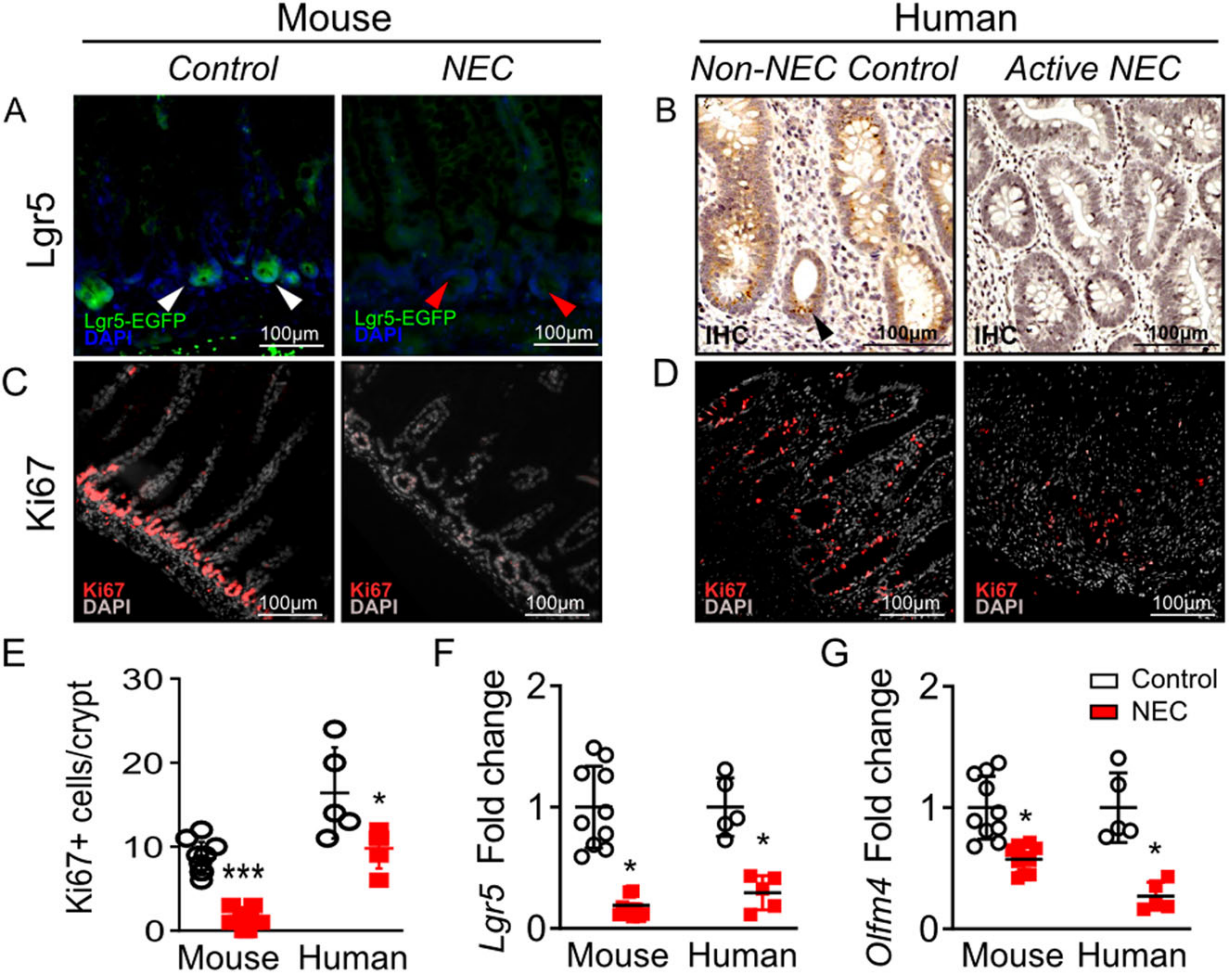
Intestinal epithelial regeneration and intestinal stem cells are impaired in both mouse and human NEC. **a** Representative immunofluorescence micrographs of EGFP + Lgr5 cells taken from terminal ileum sections of Lrg5-EGFP “knock-in” mice (white arrow heads indicate positive cells; red arrow heads indicate no positive cells). **b** Representative immunohistochemistry micrographs of terminal ileal human NEC and non-NEC control stained for Lgr5 (black arrow heads indicate positive cells). Representative immunofluorescence micrographs of terminal ileum sections stained for Ki67 in mouse (**c**) and human (**d**) with NEC. **e** Quantification of Ki67 positive cells per crypts from all experimental groups. Relative gene expression for ISC markers (**f**) *Lgr5*, and (**g**) *Olfm4*. Samples were taken from the terminal ileum of each group. Data are presented as means ± SD. *p < 0.05; **p < 0.01, using one-way ANOVA with post hoc tests

To study the intestinal epithelial regeneration during NEC, we quantified intestinal stem cells and epithelial cell proliferation. Both mouse and human NEC ileum exhibited a profound decrease in the expression of Lgr5 positive ISC at the crypts relative to control (Fig. 1a, b). The number of Ki67 positive proliferating epithelial cells in the NEC ileum was also significantly reduced compared to control groups (Fig. 1c–e). In addition, both mouse and human NEC ileum showed decreased gene expression of the ISC markers *Lgr5* and *Olfm4* (Fig. 1f, g). Collectively, these findings demonstrate that severe gut injury in NEC is associated with a reduction in ISC and is accompanied by poor intestinal regeneration.

### Activity of endogenous Wnt signaling is impaired in mouse and human NEC

**Fig. 2:**
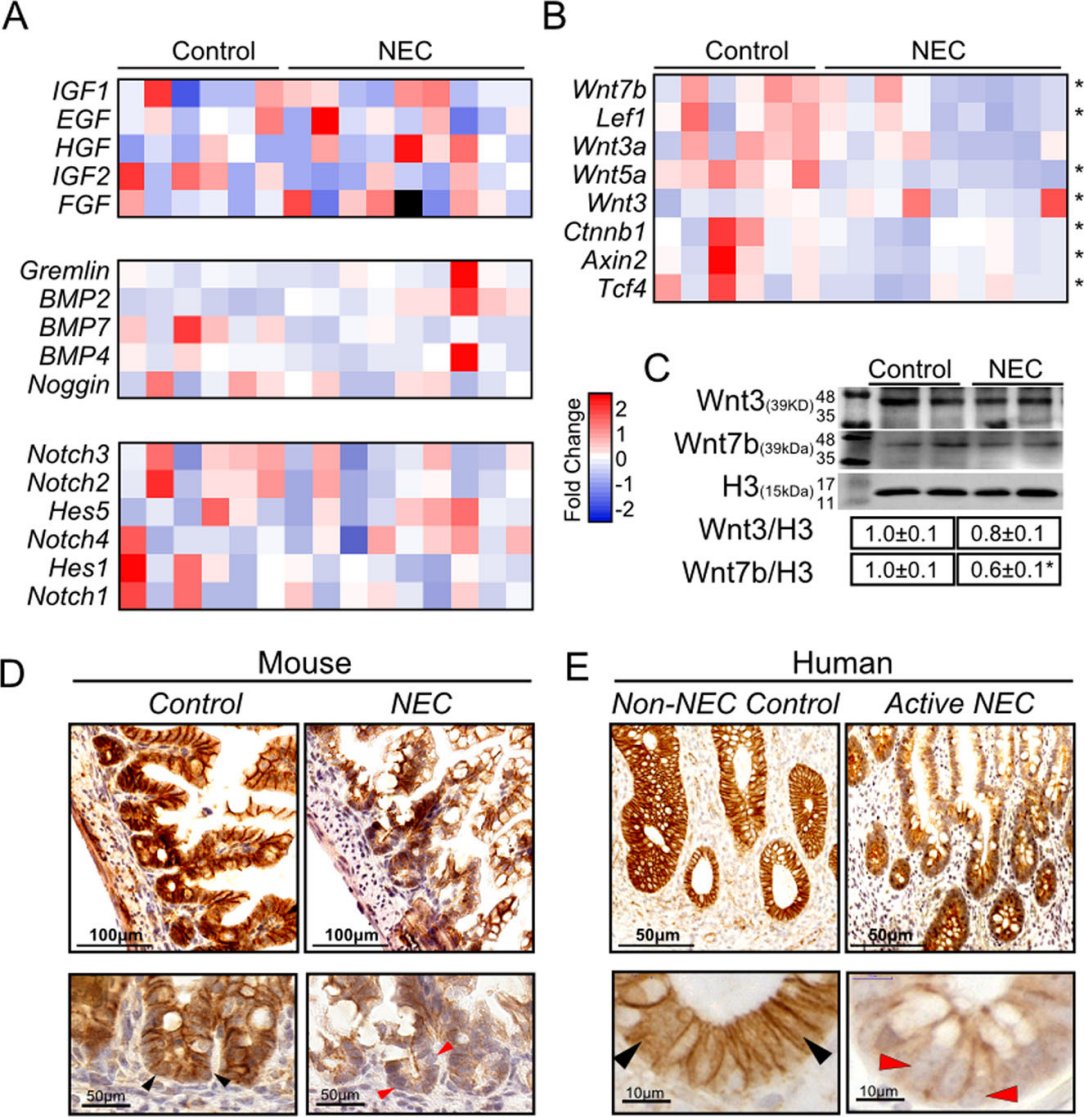
Activity of endogenous Wnt signalling in the intestinal epithelium is impaired in mouse and human NEC. **a** Expression profiles of genes involved in growth factors, BMP, Notch, and **b** Wnt transcript levels in the intestinal epithelium. Data is normalized to control. **c** Representative western blot images and quantification of Wnt3 and Wnt7b protein from each group. **d** Representative micrographs of immunohistochemical β-catenin staining for each group in mouse NEC (black arrow heads indicate nuclear translocation of β-catenin, red arrow heads indicate no nuclear β-catenin). **e** Representative micrographs of immunohistochemical β-catenin staining for ileum in human NEC and non-NEC controls (black arrow heads indicate nuclear translocation of β-catenin, red arrow heads indicate absent nuclear β-catenin). Data are presented as means ± SD. *p < 0.05; **p < 0.01, using one-way ANOVA with post hoc tests

Several pathways, including growth factors, BMP, Notch, and Wnt signaling have previously been shown to regulate ISC expression and function^13,33^. Analysis of transcription demonstrated that expressions of major genes in the BMP and Notch signaling pathways, and several growth factors remains similar in NEC mouse ileum relative to control (Fig. 2a). By contrast, a decrease in Wnt pathway major genes was observed in ileal samples of mice with experimental NEC relative to control mice (Fig. 2b). Wnt3 is released by mature epithelial Paneth cells and is essential for promoting intestinal stem cell activation after injury^34^. Paneth cells are still premature in the neonatal mouse intestine during NEC induction, no change of Wnt3 expression was observed in the NEC intestine; however, Wnt7b protein levels were significantly decreased in NEC (Fig. 2c). Accompanying the decrease in Wnt7b protein we observed in NEC, Wnt signaling, assessed via activated nuclear β-catenin, was also decreased at the bottom of crypts, where the intestinal stem cells reside, in both with experimental NEC and acute active human NEC (Fig. 2d, e). These data suggest that the NEC induced impairment of intestinal regeneration is due to deficient activity of the Wnt pathway.

### Organoids derived from NEC damaged intestine failed to maintain epithelial balance of proliferation and differentiation, but were rescued by Wnt7b supplementation

**Fig. 3:**
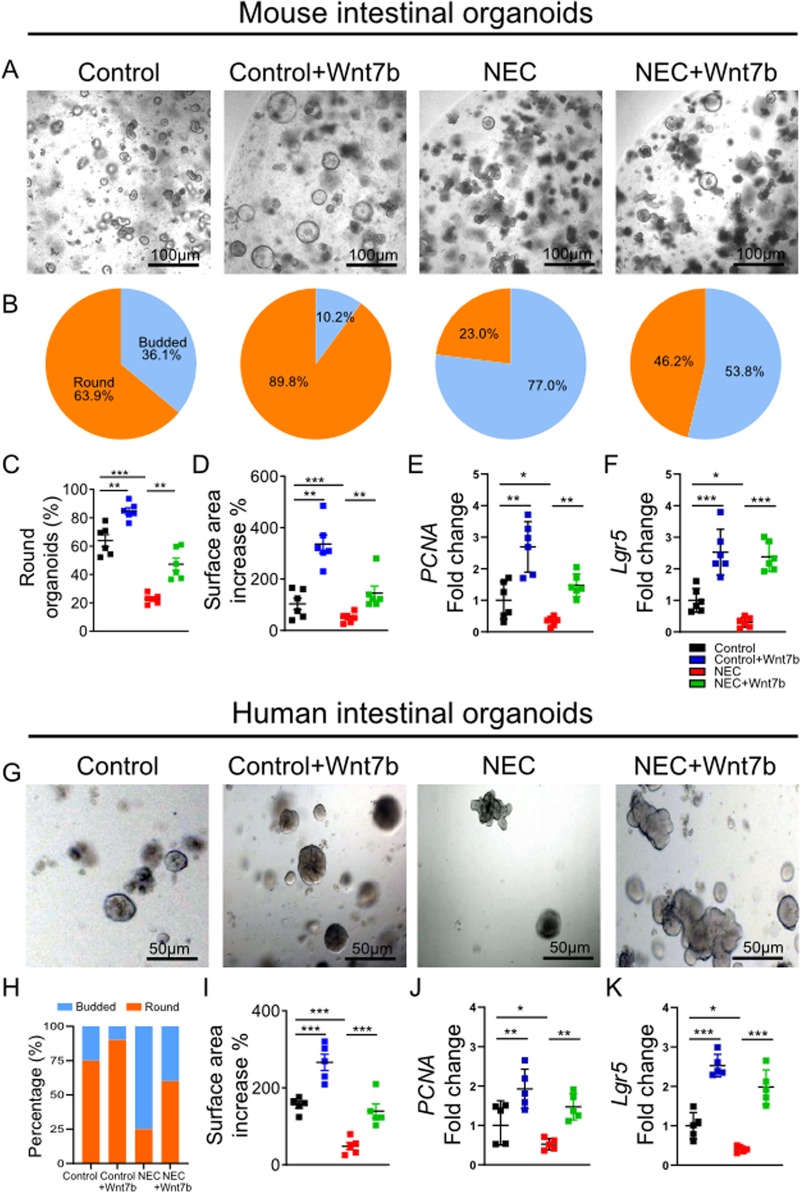
Organoids derived from NEC damaged intestine failed to maintain epithelial balance of proliferation and differentiation, but were rescued by Wnt7b supplementation. **a** Representative micrographs of mouse intestinal organoids of control, control + Wnt7b, NEC, and NEC + Wnt7b. **b** Round compared to budded mouse intestinal organoids with percentages for all experimental groups. **c** Round organoid percentage for all experimental groups. **d** Surface area increase as a percentage for all experimental groups. Relative gene expression of **e** PNCA and **f** Lgr5 for each mouse intestinal organoid group. **g** Representative micrographs of human intestinal organoids of control, control + Wnt7b, NEC, and NEC + Wnt7b. **h** Percentage of budded and round human intestinal organoids for all. Relative gene expression of **i** PNCA and **j** Lgr5 for human intestinal organoid group. Data are presented as means ± SD. *p < 0.05; **p < 0.01; ***p < 0.001, using one-way ANOVA with post hoc tests

Intestinal organoids are a robust model to investigate intestinal regeneration and to screen for therapeutic interventions^22^. First, we generated intestinal organoids from mice with NEC. NEC-derived organoids compared to those derived from controls were smaller indicating less proliferation and had more budding indicating more differentiation (Fig. 3a–d). There was also lower expression of proliferation marker *PCNA* and ISC marker *Lgr5* in NEC organoids (Fig. 3e, f). In addition, we cultured the control organoids in medium with Wnt or without Wnt. The organoids in the group with -Wnt medium had reduced proliferation (smaller size) and increased differentiation (more budding) compared to the organoids in +Wnt medium. This is a similar growth response to what was observed in organoids derived from NEC tissue (Supplementary Fig. 1). These findings indicated that Wnt deficiency can lead to NEC-like injury in organoids.

Within the Wnt pathway, we found that Wnt7b was the most decreased factor in the NEC group relative to control in above experimental NEC study. Therefore, we investigated if restoration of Wnt7b through supplementation would rescue intestinal regeneration ability in our organoids model. Since the intestinal organoid culture medium already includes Wnt to maintain growth and survival^35^, we studied the impact of Wnt7b by adding Wnt7b (200 ng/ml) to the medium. Administration of Wnt7b increased the NEC injured organoid surface area and prevented organoid budding (Fig. 3a–d). Wnt7b treatment maintained intestinal proliferation and ISC in the organoids derived from experimental NEC (Fig. 3e, f). Similar to the organoids derived from mice with NEC, the organoids derived from human infants with NEC behaved differently from those derived from controls. NEC organoids from both mice and humans were smaller in size suggesting decreased proliferation and had more budding, suggesting increased differentiation. In addition, human NEC organoids expressed less proliferation marker *PCNA*, and less ISC marker *Lgr5* (Fig. 3g–k). Wnt7b administration recued these changes in NEC to the level seen in control (Fig. 3g–k). Overall, organoids derived from NEC damaged intestine failed to restore the balance of proliferation and differentiation, while Wnt7b promoted the increase of intestinal stem cells and maintained intestinal proliferation in organoids derived from human and mouse NEC.

### Wnt7b administration attenuates intestinal injury by rescuing intestinal stem cells and restoring intestinal regeneration in experimental NEC

**Fig. 4:**
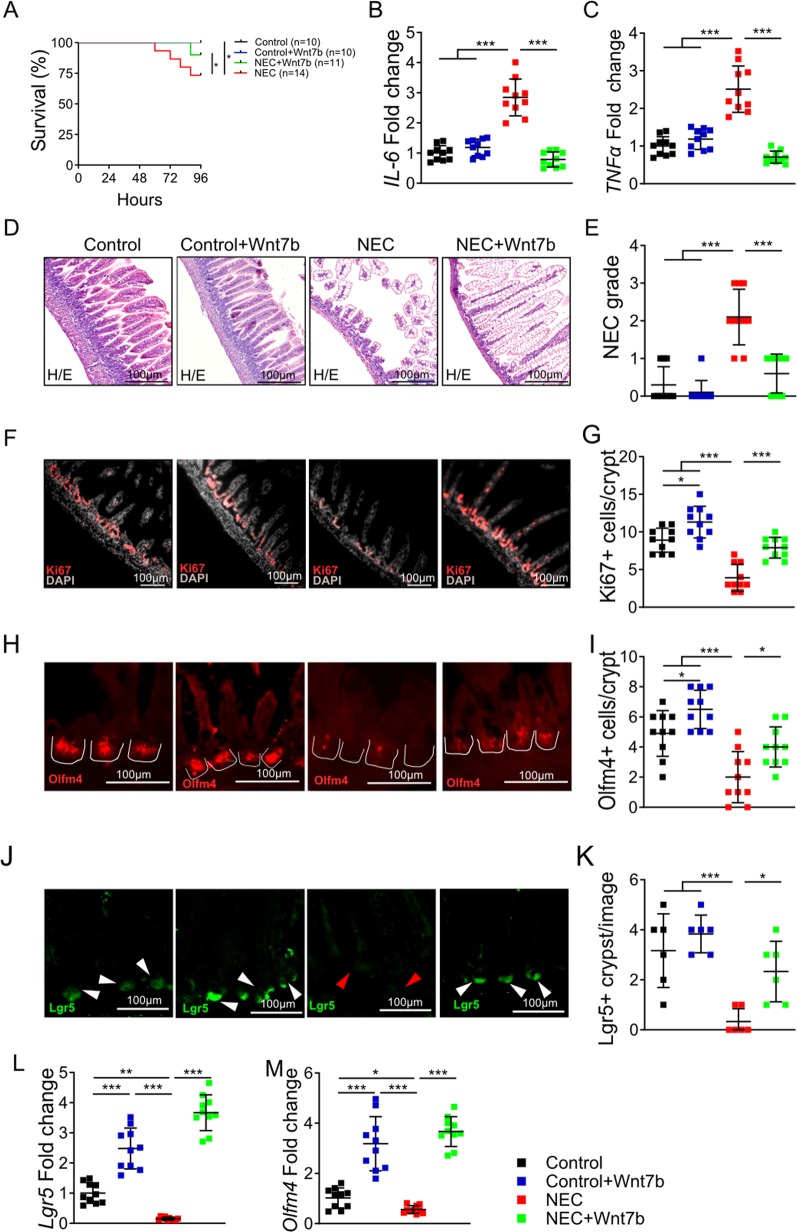
Wnt7b administration attenuates intestinal injury by rescuing intestinal stem cells and regeneration in mouse NEC. **a** Survival curves for C57/Bl6 mice from control (*n* = 10), control + Wnt7b (*n* = 10) NEC (*n* = 14), and NEC + Wnt7b (*n* = 11) groups. Relative gene expression of **b** *IL-6*, and **c** *TNFα* in the terminal ileum from each Control, Control + Wnt7b, NEC, and NEC + Wnt7b group. **d** Representative H&E-stained histomicrographs of the terminal ileum from each experimental group. **e** NEC severity scores graded by analysis of H&E histomicrographs. **f** Representative immunofluorescence micrographs of terminal ileum sections stained for Ki67 from each experimental group. **g** Quantification of Ki67 + cells per crypt for each group. **h** Representative immunofluorescence micrographs of terminal ileum sections stained for Olmf4 from each experimental group (white lines indicate crypts). **i** Quantification of Olfm4+ cells per crypt for each group. **j** Representative immunofluorescence micrographs of EGFP + Lgr5 cells taken from terminal ileum sections of Lrg5-EGFP “knock-in” mice (white arrow heads indicate positive cells; red arrow heads indicate no positive cells). **k** Quantification of Lgr5+ cells per crypt per image for each group. Relative gene expression of *Lgr5* (**l**), and *Olfm4* (**m**) in the terminal ileum from each experimental group. Data are presented as mean ± SD. *p < 0.05; **p < 0.01; ***p < 0.001, using one-way ANOVA with post hoc tests

We studied the effects of Wnt7b on intestinal injury in experimental NEC. Wnt7b was administered to mice pups during NEC induction or to the control breastfed pups. An improvement was observed in the survival of the Wnt7b administered NEC group (*n* = 11) compared to the NEC alone group (*n* = 14) (Fig. 4a). NEC induced inflammation (*IL-6*, *TNFα*), was decreased after Wnt7b treatment (Fig. 4b, c). NEC-like intestinal injury (histological grade ≥ 2) was present in 8/10 pup mice, with 5/10 developing moderate and 3/10 severe damage. These changes, occurring in a great proportion of the pup mice exposed to NEC, indicate great efficiency of our model in inducing the disease. However, NEC induced intestinal morphological injury was rescued by Wnt7b treatment (Fig. 4d, e). Intestinal epithelial proliferation was reduced in NEC, but was rescued by Wnt7b administration (Fig. 4d, e). Proliferation marker *Ki67* was significantly reduced in NEC relative to control, and was restored with Wnt7b administration (Fig. 4f, g). Furthermore, intestinal stem cell markers *Lgr5* and *Olfm4* were increased in the Wnt7b treatment group compared to the NEC alone group (Fig. 4h–m). Collectively, these findings suggest that Wnt7b reduced the mortality and severity of NEC through increasing intestinal regeneration.

## Discussion

**Fig. 5:**
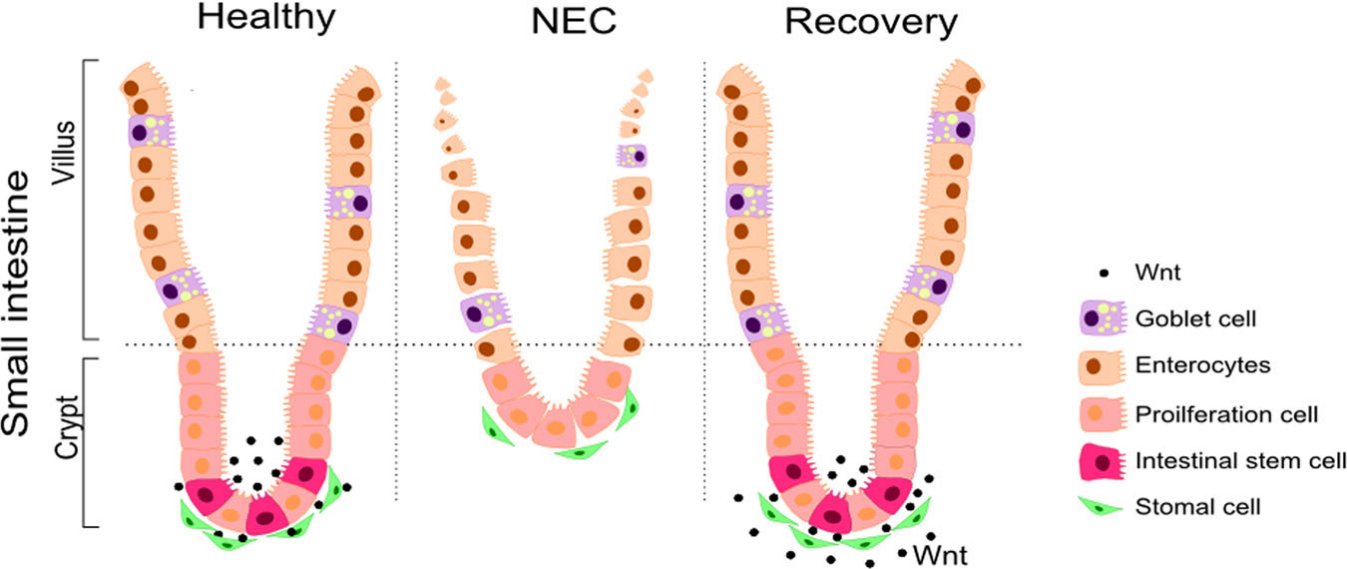
Schematic diagram illustrating the role of Wnt/ β-catenin in intestinal regeneration in NEC. Epithelial regeneration is crucial for intestinal recovery from injury which is facilitated by intestinal stem cells that are activated by the Wnt/β-catenin pathway. In both experimental and human NEC, intestinal stem cells and epithelial regeneration are impaired due to deficiency of Wnt/β-catenin. Administration of exogenous Wnt recombinant protein restores intestinal regeneration and attenuates NEC injury

In this study, we demonstrated that reduced Wnt activity is present in neonatal mice with NEC, resulting in impaired ISC and ISC-mediated epithelial regeneration. These findings were validated in human preterm infants by studying the intestinal epithelium during acute active NEC. These alterations can contribute to the severe mucosal damage which characterizes the disease. We also showed that provision of an exogenous source of Wnt, activated ISC to promote epithelial cell proliferation, differentiation, and repair of the intestinal epithelial injury (Fig. 5).

The gut epithelium undergoes self-renewal and regeneration, and ISC proliferation is essential for restoring the epithelial layer after intestinal insult^7^. Active ISC maintain the homeostatic regenerative capacity of the intestine, and in this study, we have shown that mitotically active *Lgr5* and *Olfm4* ISC are impaired in NEC. Available evidence in the literature supports the requirement of restoring activation of ISC and promoting intestinal cell proliferation to counteract intestinal injury related to NEC. For example, heparin-binding EGF-like Growth Factor reduces NEC-induced intestinal injury via its ability to protect ISC^36^. Retinoic acid prevents and treats NEC by modulating the ISC pool within the small intestine^11^. In addition, breast milk-derived exosomes stimulate ISC activity, enhance proliferation of IEC-18 cells, and thus provide an alternative preventative method for infants at high risk of developing NEC when mother’s milk is not available^37^. Taken together, our findings demonstrate that dysfunction of ISC is associated with NEC development in preterm infants and supports the notion that restoring ISC is a promising therapeutic target for intervention against NEC.

Several signaling pathways, including the Wnt/β-catenin, BMP, growth factors and Notch cascades are critical for ISC self-renewal and proliferation^13,33^. We found that the Wnt and β-catenin pathways were dysregulated in organoids, in experimental NEC and most importantly in human NEC. This deregulation leads to impairment of intestinal epithelial stem cell proliferation and differentiation. Sodhi et al. also showed that NEC-induced inhibition of β-catenin signaling leads to reduced enterocyte proliferation in response to TLR4 activation^38^. In our experiments, we performed immunohistochemistry staining of β-catenin in mice and humans with NEC. This revealed significantly decreased β-catenin expression in the intestinal crypts leading to impairment of intestinal stem cells. NEC results in apoptosis of endogenous Wnt-releasing cells, such as Paneth^18^ and stromal cells^19–21^, leading to a reduction in Wnt expression and secretion. In these circumstances, an exogenous source of Wnt may promote intestinal regeneration and thereby serve as a novel treatment for NEC. Although Wnt7b has not been extensively characterized in the intestine, we tested the role of Wnt7b administration and found compelling evidence for its beneficial effects in promoting intestinal growth and attenuating the intestinal injury induced by NEC. Various studies implicate Wnt7b in epithelial development and regeneration in the lung^39^, pancreas^40^ and kidney^41^. Interestingly, Wnt7b is activated in skin wounds and results in regeneration through Prostaglandin E2 (PGE2)^42^, which is the main downstream product of cyclooxygenase-2 (COX-2). Both COX-2 and its prostaglandin products are important factors in gut homeostasis and inflammation during NEC^43,44^, and the beneficial effect mediated by Wnt signaling may be acting upstream to the COX-2/PGE2 pathway. Wnt signaling is involved in multiple biological processes^45^ and its constitutive activation of Wnt might promote cell proliferation and tumor formation^46^. Thus, a thorough evaluation of routes and dosages and potential side effects of Wnt administration is required for translation of our findings into a clinical setting. Various ongoing clinical trials using Wnt agonists provide insight into the viability of Wnt-based treatment^47^. Transient and controlled activation of Wnt should be further investigated as a novel treatment of NEC.

Organoids are a dynamic model which help develop our understanding of intestinal pathophysiology in NEC and allow for various therapeutic treatment interventions. Intestinal organoid formation and maintenance requires several biological processes which include stem cell proliferation, self-renewal and differentiation, and new crypts formation (budding)^48^. During intestinal epithelial repair, cell proliferation in the crypts, triggered by Lgr5+ stem cell stimulation, leads to formation of more cells, and differentiation into functionally mature cells which project out from the organoids to form new buds^13,48,49^. Intestinal homeostasis and balance between cell proliferation and differentiation is crucial to maintain organoid viability. In the current study, we successfully derived intestinal organoids from both mice and human NEC. We discovered that NEC-derived organoids were unable to maintain intestinal homeostasis due to reduced stem cell proliferation and a higher propensity to differentiate and bud. These findings align with our observations in NEC ileum where there were also reduced stem cell proliferation and regeneration. Furthermore, using NEC-derived organoids we confirmed that exogenous Wnt administration restored the organoids intestinal homeostasis supporting the novel potential therapeutic role of exogenous Wnt.

Our experimental model induced morphological changes similar to NEC in a great proportion (80%) of the animals. The variability in individual response to the stress factors used for NEC induction is quite limited but can explain some of the differences observed in gene expression.

In summary, our findings indicate that NEC induces disruption of the intestinal Wnt-ISC-regeneration axis and exogenous Wnt administration activated ISC to promote epithelial cell proliferation, differentiation, and repair of the injured intestinal epithelium. This study provides an extensive characterization of the pathogenesis of NEC not only experimentally but also in human neonates and demonstrates the value of a potentially novel therapeutic strategy for preterm infants with NEC.

## Supplementary information

Supplementary Data
