# Supplementary material for: Impaired Wnt/β-catenin pathway leads to dysfunction of intestinal regeneration during necrotizing enterocolitis
Source: Cell Death Dis. 2019 Oct 3;10(10):743. doi: 10.1038/s41419-019-1987-1 (PMC6776513; doi:10.1038/s41419-019-1987-1)
Supplement: Supplementary file 1 — Supplementary Data [file 41419_2019_1987_MOESM1_ESM.docx]

**Supplementary Data:**

**
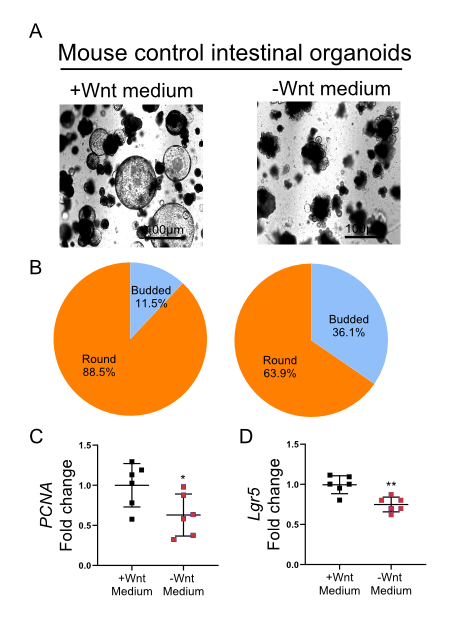
**

**Supplementary Figure 1: Wnt deficiency leads to NEC-like injury in organoids culture**

**(A)** Representative micrographs of mouse intestinal organoids with +Wnt and -Wnt medium. **(B)** Percentage of round and budded mouse intestinal organoids in both groups. Relative gene expression of **(C)** PNCA and **(D)** Lgr5 for both groups. Data are presented as means ± SD. *p < 0.05; **p < 0.01, using one-way ANOVA with post-hoc tests.
